# The efficiency of a clinical pathway to guide combined applications of interventional pulmonology in undiagnosed pleural effusions

**DOI:** 10.1038/s41598-022-15454-6

**Published:** 2022-07-01

**Authors:** Yuan Liu, Lili Geng, Jian Xu, Mei Sun, Na Gao, Jing Zhao, Xue Han, Xiaolin Zhang, Xiaohui Zhao, Ling Jiang, Junjun Zhao

**Affiliations:** 1 Department of Respiratory and Critical Care Medicine, Dalian Municipal Central Hospital Affiliated of Dalian Medical University, Number 826, XiNan Road, Dalian, 116033 Liaoning Province China; 2 Department of Digestive System, Dalian Municipal Central Hospital Affiliated of Dalian Medical University, Dalian, 116033 China; 3 Department of Pathology, Dalian Municipal Central Hospital Affiliated of Dalian Medical University, Dalian, 116033 China

**Keywords:** Respiratory tract diseases, Medical research

## Abstract

The diagnostic procedure of pleural effusion (PEs) is challenging due to low detection rates and numerous aetiologies. Hence, any attempt to enhance diagnosis is worthwhile. We present a clinical pathway to guide combined application of interventional pulmonology (IP) for tracing causes of undiagnosed PEs. Subjects with undiagnosed PEs were identified in the Hospital Information System of Dalian Municipal Central Hospital from January 1, 2012, to December 31, 2018. Eligible subjects were divided into a group of combined tests and a group of medical thoracoscopy (MT). Optimal and subsequent diagnostic tests were performed depending on the guidance of the clinical pathway by matching profitable chest lesions with the respective adaptation. As the guidance of clinical pathway, common bronchoscopy would be preferentially selected if pulmonary lesions involved or within the central bronchus, EBUS-TBNA was favoured when pulmonary lesions were adjacent to the central bronchus or with the enlarged mediastinal/hilar lymph nodes, guided bronchoscopy would be preferred if pulmonary nodules/masses were larger than 20 mm with discernible bronchus signs, CT-assisted transthoracic core biopsy was preferred if pulmonary nodules were less than 20 mm, image guided cutting needle biopsy was the recommendation if the pleural thickness was larger than 10 mm and pulmonary lesions were miliary. MT was preferred only when undiagnosed PEs was the initial symptom and pulmonary lesions were miliary or absent. A total of 83.57% cases of undiagnosed PEs were eligible for the clinical pathway, and 659 and 216 subjects were included in the combined tests and MT groups, respectively, depending on the optimal recommendation of the clinical pathway. The total diagnostic yields in the combined tests and MT groups were 95.99% and 91.20%, respectively, and the difference in total diagnostic yield was statistically significant (χ^2^ = 7.510, *p* = 0.006). Overall, clinical pathway guidance of the combined application of IP is useful for tracing the causes of undiagnosed PEs. The diagnostic yield of undiagnosed PEs is significantly increased compared with that of MT alone.

## Introduction

Pleural effusions (PEs), which are mediated by a variety of mechanisms and related to increased production and reduced absorption of pleural fluid (PF), arise from systemic, inflammatory, infectious and malignant conditions. Aetiologies include more than 50 types of diseases. Moreover, diagnosis is a considerable challenge due to the extensive aetiological spectrum and differential diagnosis considerations. Overall, diagnostic evaluation includes clinical examination, imaging study and PF analysis^1^. The patient’s medical history plays an important role in the evaluation, and PEs commonly occurs along with progression of prior disease^2^. A total of 75% of effusions might be diagnosed before analysis of PF because their causes may be attributed to chronic heart failure (CHF), pneumonia or malignancy^3^. Analysis of PF provides additional information in which medical history and clinical information do not interpret causes. The most transudative effusions are complications of CHF, with exudative effusions being complications of pneumonia^4^. Although microbiological identification of a pathogenic organism can confirm infective PEs, only 25% of the underlying pathogen is identified by Gram staining of a non-purulent parapneumonic effusion^5^. The yield of PF culture has a sensitivity of 10–20%^6^. The presence of acid-fast bacilli in PF is only 7%^7^. The diagnostic yield of PF cytology is also low^8^, and rates of overall sensitivity and specificity for malignant pleural effusions (MPE) of 69.4% and 93.3%, respectively, have been found^9^. Some diagnostic biomarkers in PF might indicate the presence of malignancy or tuberculous pleurisy^10,11^, though confirmation by microbiology, cytology or histopathology in suspected PEs cannot be replaced by diagnostic biomarkers^7,12^. As the incidence of undiagnosed PEs is 20% despite enhanced detection sensitivity^6^, further pathological examination is needed to trace its causes.

Pleural tissue biopsies are usually recommended for undiagnosed PEs^3,4,6,13–15^. Blind, image-guided needle biopsy, medical thoracoscopy (MT), video-assisted thoracic surgery (VATS) and pleuroscopy are procedures that provide access to the pleural space, but with unequal invasive injury^16,17^. However, the field of interventional pulmonology (IP) has experienced significant growth in recent years, and interventional bronchoscopy has advanced the evaluation and management of central airway obstruction, mediastinal/hilar adenopathy and lung nodules/masses^18,19^. Endobronchial ultrasound-guided transbronchial needle aspiration biopsy (EBUS-TBNA) is more suitable for the diagnosis of central bronchus, mediastinal and hilus lesions under direct real-time endobronchial ultrasound guidance^20^. Several guided bronchoscopy technologies, including electromagnetic navigation bronchoscopy (ENB), virtual bronchoscope navigation (VBN), radial endobronchial ultrasound (R-EBUS), ultrathin bronchoscopy, and guide sheaths, improve the rate of peripheral nodule detection^21^. The pooled diagnostic yield of guided bronchoscopy is 70%, higher than that for traditional transbronchial biopsy^21^. Image guidance increases the yield of cutting needle biopsy (CNB) and reduces complication rates in the presence of pleural thickening^22^. The diagnosis based on interventional bronchoscopy in pulmonary lesions might indicate concurrent PEs as approximately 50% to 65% of MPE is secondary to metastases from lung and breast cancer^14^.

**Figure 1:**
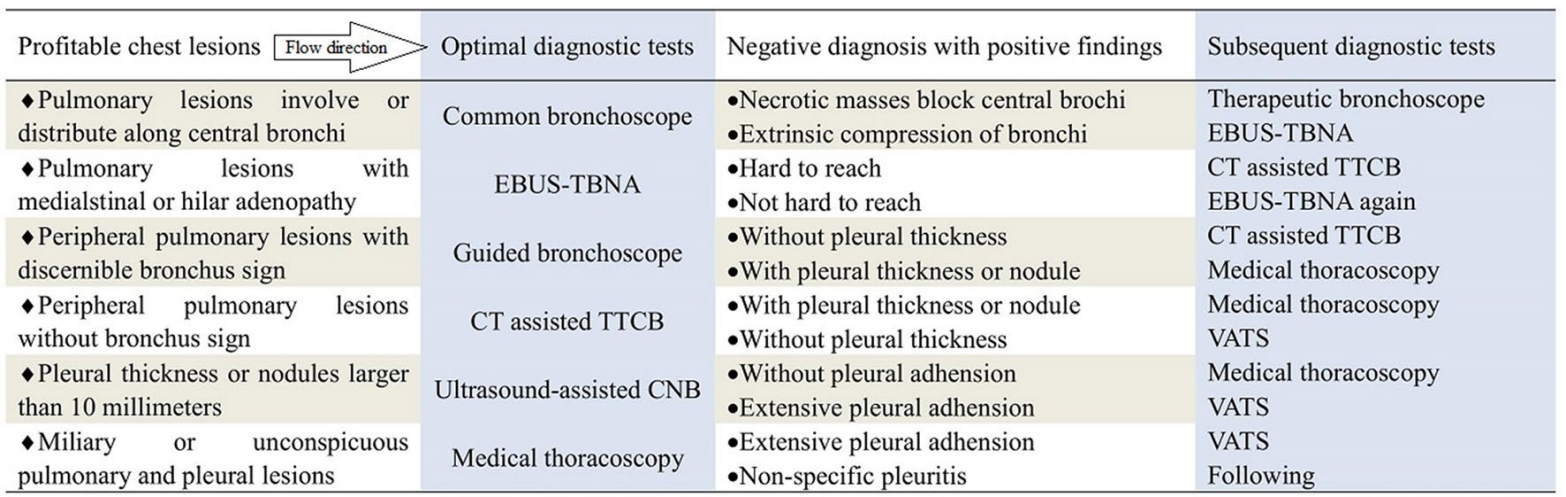
A clinical pathway to guide the selection of optimal and subsequent diagnostic tests. The optimal diagnostic tests in the first grey column were selected depending on the features of chest lesions in the corresponding line of the previous column. The subsequent diagnostic tests in the second grey column were selected depending on the findings of optimal diagnostic tests. *EBUS-TBNA* endobronchial ultrasound-guided transbronchial needle aspiration biopsy, *TTCB* transthoracic core biopsy, *CNB* cutting needle biopsy, *VATS* video-assisted thoracoscopic surgery.

In our hospital, the technique of IP has been applied for nearly 20 years to diagnose and manage pulmonary lesions. A clinical pathway have been performed to guide application of international bronchoscopy, MT and needle biopsy for the purpose of regulating the application of every technique of IP by matching chest lesions with diagnostic adaptations of every technique since Jan 2012 (Fig. 1). Almost thousand cases of interventional bronchoscopy, MT and needle biopsy are performed per year for diagnosis of pulmonary lesions by guidance of this clinical pathway since 2012. PEs is the most common concurrent condition with chest lesions. So, we searched in our hospital databases using pleural effusions as the keyword and explored the efficiency of combined application of IP to trace causes of undiagnosed PEs and evaluated the efficiency of clinical pathway under its guidance.

## Results

### PEs clinical information

**Figure 2:**
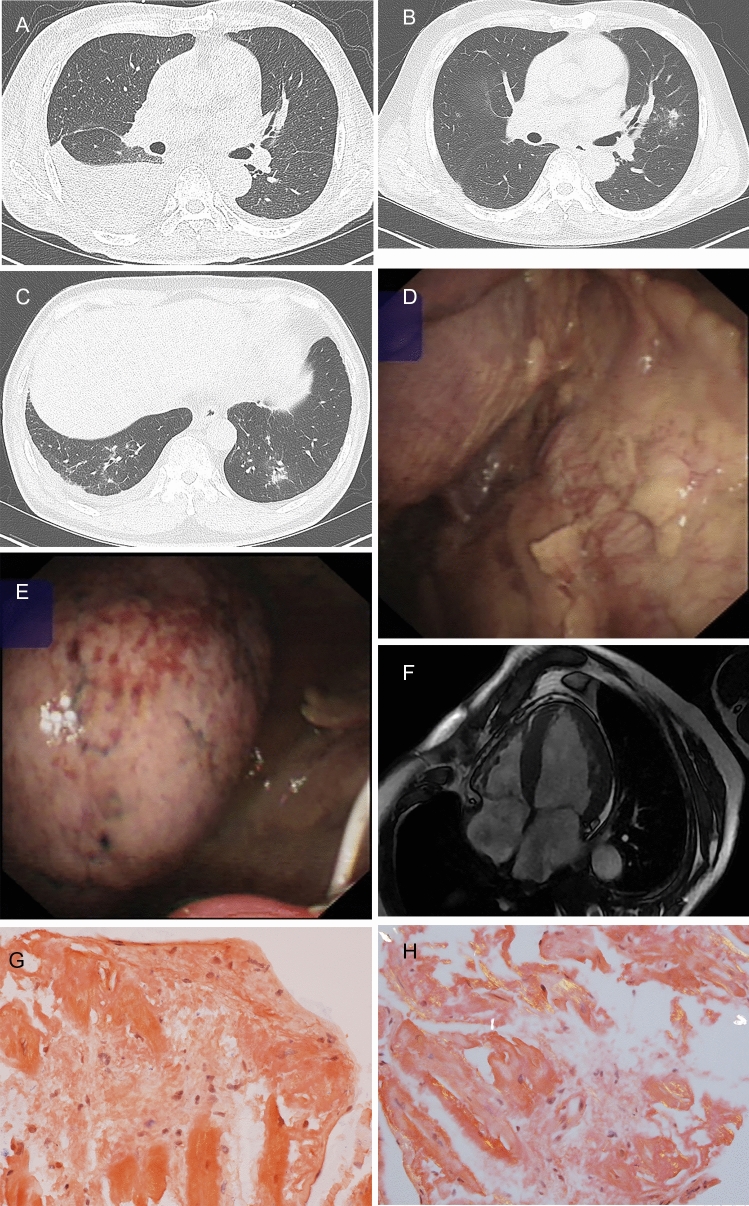
A primary systemic amyloidosis with transudative pleural effusions. Bilateral pleural effusions and prominent lesions on the right side were shown on images of the lung window (**A**). New lesions on the bilateral pulmonary field were identified as oedema after diagnostic thoracentesis and drainage (**B**, **C**). Oedema and hyperaemia on the pleura of the diaphragmatic surface (**E**) and irregular plaque of the parietal pleura near the costophrenic angle (**D**) were shown by medical thoracoscopy, and amyloidosis was suspected by histopathology. Diffuse left ventricular hypertrophy and prominent interventricular septum were shown by magnetic resonance imaging (**F**), and heart valve regurgitation existed. Cardiac amyloidosis was identified by myocardial biopsy (**G**, **H**), and amyloid-associated protein was deposited in the myocardial intercellular space and subendocardial layer on pathological slices (Congo red staining, 200 times magnification).

A total of 7657 subjects with new PEs were identified in the Hospital Information System (4738 males and mean age, 67.73 ± 18.89 years). Of them, 5628 (73.50%) could be diagnosed by clinical evidence, and 2029 (26.50%) showed no clinical evidence. Except for 268 subjects, diagnostic thoracentesis was performed in 1761 subjects (supplemental Table 1). Exudative and transudative effusions were identified in 1580 (89.79%) and 181 (10.21%) subjects, respectively. Except for one subject because of abundant mesothelial cells in the PF, 99.45% (180/181 cases) of transudative effusion cases had clinical evidence, and primary systemic amyloidosis was suspected by MT (Fig. 2). A total of 30.00% (474/1580 cases) of exudative effusion cases were diagnosed by PF testing, whereas 70.00% (1106/1580 cases) of subjects were not diagnosed. Finally, 14.89% (1107/7389 cases) [95% confidence interval: 14.09%, 15.69%] of PEs was consistent with undiagnosed PEs including 1106 cases of exudative effusion and one case of transudative effusion.

### The enrolment rate of the clinical pathway for undiagnosed PEs

**Figure 3:**
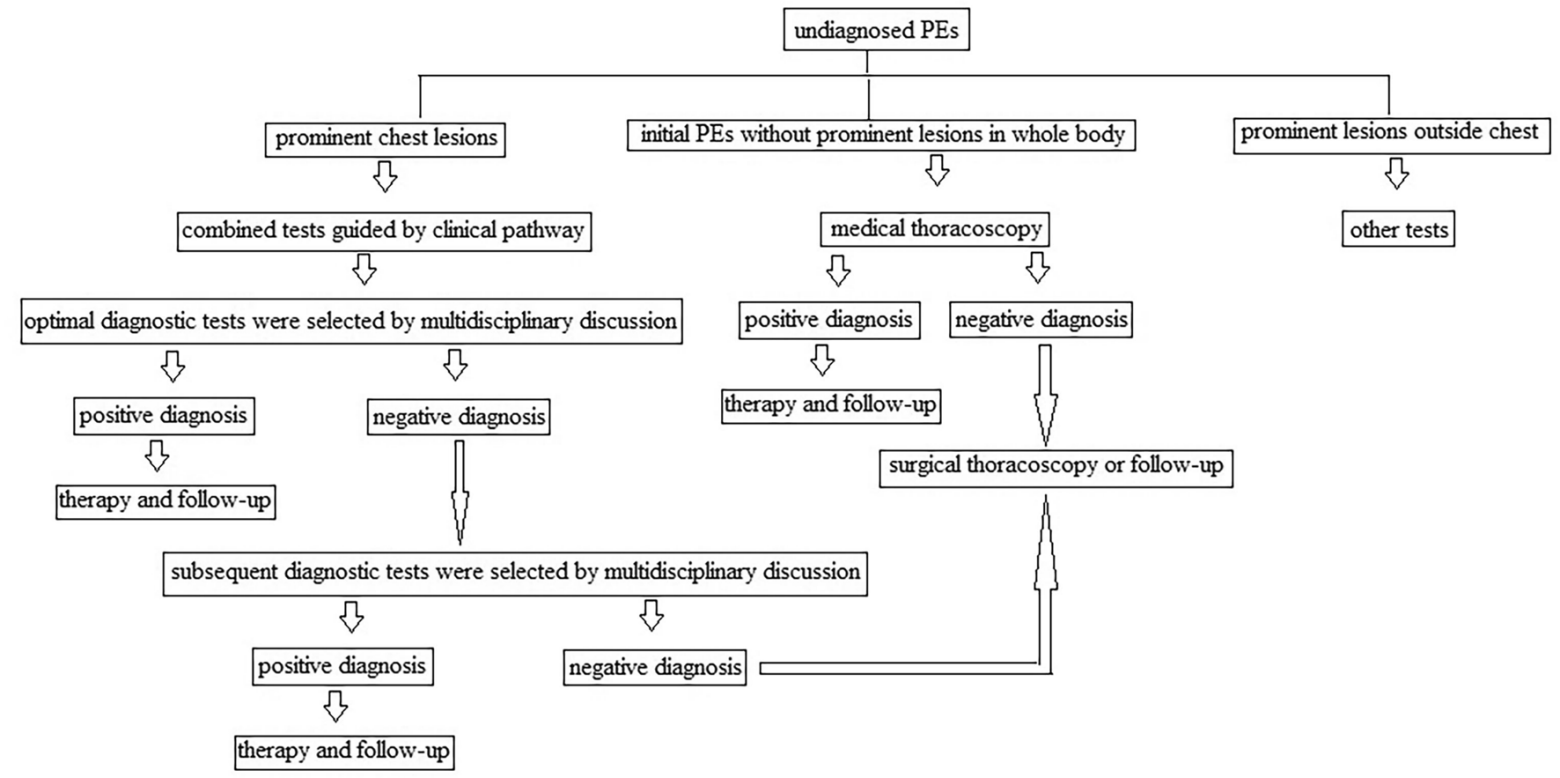
A flow chart for enrolling clinical pathway to diagnose undiagnosed Pes.

In total, 60 subjects refused, and 16.43% of subjects (172/1047 cases) could not be enrolled by the clinical pathway because of lesions outside the chest and other tests being optimal (supplemental Table 1). Ultimately, 875 subjects were eligible (577 males and mean age, 64.52 ± 14.30 years), and the enrolment rate of the clinical pathway was 83.57% (875/1047 cases) [95% confidence interval: 81.32%, 85.82%]. A flow chart of enrolling clinical pathway was shown in Fig. 3. Among the 875 enrolled subjects, 659 were included in the group of combined tests because interventional bronchoscopy or needle biopsy was preferred under the guidance of the clinical pathway (supplemental Table 2). The other 216 subjects were included in the group of MT because pleural biopsy by MT was preferred under the clinical pathway guidance (supplemental Table 3).

### The diagnostic yield of MT

A total of 88.89% (192/216 cases) [95% confidence interval: 84.70%, 93.08%] of undiagnosed PEs cases in the MT group were diagnosed by optimal choice tests under the guidance of the clinical pathway (supplemental Table 3). Five negative subjects subsequently underwent VATS, and all of the final diagnoses had been listed in supplemental Table 3. The total diagnostic yield reached 91.20% (197/216 cases) [95% confidence interval: 87.44%, 94.96%]. The difference between the optimal and total diagnostic yields was not statistically significant (Table 2).

### The diagnostic yield of combined tests

The optimal choice diagnostic tests and respective diagnostic yield in the group of combined tests under the guidance of the clinical pathway are listed in Table 1. Although the respective indications of diagnostic tests varied for undiagnosed PEs, the diagnostic yield of MT was not significantly higher than that of EBUS-TBNA, guided bronchoscopy or pleural CNB (Table 1). Nevertheless, the diagnostic yield of MT was significantly higher than that of common bronchoscopy and lower than that of transthoracic core biopsy (TTCB). The diagnostic yield of optimal choice in the group of combined tests was 83.76% (552/659 cases) [95% confidence interval: 80.94%, 86.58%], and the diagnostic yield of MT did not significantly differ from that of combined tests (Table 2).

**Table 1:** Diagnostic yield of optimal inspective tests by guidance of the clinical pathway.

Tests type of initial choice	Included cases	Positive cases	Diagnostic yield (%)	95% confidence interval (%)	χ^2^	*p*
Common bronchoscopy	282	206	73.05	67.88, 78.22	19.121	*0.000*
EBUS-TBNA	145	132	91.35	86.78, 95.92	0.434	0.510
Guided bronchoscopy	65	55	84.62	75.86, 93.38	0.851	0.354
Pulmonary TTCB	145	139	95.86	82.63, 99.09	5.537	*0.019*
Pleural CNB	22	20	90.91	78.90, 102.92	0.084	0.772
Medical thoracoscopy	216	192	88.89	84.70, 93.08		

The guiding techniques include radial endobronchial ultrasound, virtual bronchoscope navigation and fluoroscopy in guided bronchoscopy. TTCB denotes chest CT-assisted transthoracic core biopsy. CNB denotes ultrasound assisted cutting needle biopsy. The diagnostic yield of MT is compared with various interventional tests by the Chi-square test, and Pearson χ^2^ and probability values are listed; *p* < 0.05 denotes that the difference is significant statistically.

Significant values are in italic.

**Table 2:** Difference of diagnostic yield in two groups.

Groups	Diagnostic yield of Optimal diagnostic tests	Total diagnostic yield of optimal and subsequent tests	χ^2^	*p*^1^
Combined tests	83.76% (552/659)	95.99% (622/648)	53.416	*0.000*
Medical thoracoscopy	88.89% (192/216)	91.20% (197/216)	0.646	0.422
X^2^	3.357	7.510		
*p*^2^	0.067	*0.006*		

Combined tests in the group include common bronchoscopy, fine bronchoscopy, therapeutic bronchoscopy, EBUS-TBNA, guided bronchoscopy, TTCB and CNB, among others. The guiding techniques include radial endobronchial ultrasound, virtual bronchoscopy navigation and fluoroscopy in guided bronchoscopy.

*p*^1^ was obtained by comparing optimal and total diagnostic yields within a group.

*p*^2^ was obtained by comparing optimal or total diagnostic yields between two groups.

Significant values are in italic.

There were 11 subjects who refused further tests or follow-up; 72 negative subjects underwent subsequent diagnostic tests, including 6 cases of MT and 13 cases of VATS, and 53 cases of other IP (supplemental Table 2). 70 subjects had a positive diagnosis and all of the final diagnoses had been listed in supplemental Table 2. The total diagnostic yield of the combined test was up to 95.99% (622/648 cases) [95% confidence interval: 94.48%, 97.50%], and the diagnostic yield was significantly enhanced compared to that of optimal tests because MT was included in subsequent tests (Table 2).

### The efficiency of the clinical pathway

The difference in total diagnostic yield in the combined tests (95.99%) and MT (91.20%) groups was also statistically significant (Table 2). Thus, interventional bronchoscopy and needle biopsy before MT, depending on the guidance of the clinical pathway, might significantly increase the diagnostic yield of undiagnosed PEs by MT.

## Discussion

The incidence of undiagnosed PEs was 14.89% in our research. Worldwide, approximately 20% of PE cases require pathological biopsy to trace the aetiology of undiagnosed PEs. Closed pleural biopsy (CPB) has been utilized for 60 years to obtain pleural tissue without the need for surgery, with only 40% diagnostic accuracy for MPE due to patchy pleural involvement^16^. Ultrasound-assisted CNB increases the combined yield to 90.0% for undiagnosed PEs^17^, and CT-guided pleural biopsy shows a diagnostic yield similar to that of ultrasound-guided biopsy^23^. Thoracoscopy has experienced rapid progression in recent years, and examination of pleural pathology by thoracoscopy has been the most common recommendation for the diagnosis of undiagnosed PEs. MT has been increasingly used by respiratory physicians under local sedation with spontaneous ventilation for the investigation of suspected MPE, with fewer injuries than surgical thoracoscopy^16,24^. Therefore, MT has more application than surgical thoracoscopy if therapeutic pulmonary lobectomy is not taken into account during the diagnosis of undiagnosed PEs^23^. The pooled diagnostic yield of MT for pleural malignancy is reportedly 92%^23^. For undiagnosed PEs, a diagnostic yield of 91.9% has been reported^25^, and the performance of VATS for pleural biopsy was similar to MT, with a diagnostic sensitivity of 90.1% to 92.6%^14^. In our study, the diagnostic yield of MT and pleural CNB for undiagnosed PEs was similar to the literature.

Interventional bronchoscopy has also experienced significant growth, allowing for evaluation and management of central airway obstruction, mediastinal/hilar adenopathy and lung nodules/masses^26,27^. EBUS and navigation bronchoscopy have revolutionized the diagnosis of pulmonary nodules and staging of lung cancer^28^, with more extensive application than common bronchoscopy for the diagnosis of peribronchial or peripheral pulmonary lesions. EBUS-TBNA shows a pooled sensitivity of approximately 90% for staging non-small-cell lung cancer and 85% for intrathoracic lymph nodal metastases from extrathoracic malignancies^18,29^, and there is excellent agreement (100%) between EBUS-TBNA and mediastinoscopy for mediastinal staging^20^. Moreover, guided bronchoscopy, including VBN, ultrathin bronchoscopy, ENB, guiding sheaths and radial EBUS, provides assistance in navigating towards peripheral lung lesions, with 70% of the pooled diagnostic yield for peripheral nodule sampling^30^. PEs is the most common complication of pulmonary lesions, and diagnosis based on pulmonary lesions might indicate a diagnosis of concurrent PE. Overall, interventional bronchoscopy might be useful for tracing the causes of undiagnosed PEs. Although there is no study on the application of interventional bronchoscopy for the diagnosis of PEs, bronchoscopy is recommended before MT for undiagnosed PEs^31^.

Some clinical pathways, flow charts and guidelines have been designed to indicate the diagnosis of PEs, MPE or parapneumonic effusion^4,6,14,31,32^. However, a clinical pathway for undiagnosed PEs is lacking, especially interventional bronchoscopy and needle biopsy. We present a clinical pathway to guide applications of interventional bronchoscopy, needle biopsy and MT for pulmonary lesions and concurrent PEs. First, there was a fairly good enrolment rate (83.57%), and most undiagnosed PEs was suitable for this guidance of the clinical pathway. The application of interventional bronchoscopy for undiagnosed PEs was then evaluated, and interventional bronchoscopy and needle biopsy for undiagnosed PEs had diagnostic yields for pulmonary lesions similar to those reported by the above studies. The diagnostic yields of EBUS-TBNA, guided bronchoscopy, pulmonary TTCB, and pleural CNB were 91.35%, 84.62%, 95.86% and 90.91%, respectively, in our research (Table 1). The diagnostic yield of optimal combined tests was slightly lower than MT but not significant. However, a brief recovery time from bronchoscopy allowed for subsequent diagnostic tests in a very short interval. Thus, MT maybe performed as a subsequent diagnostic test in negative subjects after optimal tests. The diagnostic yield would be significantly enhanced depending on this guidance of the clinical pathway to perform interventional bronchoscopy or needle biopsy first and then MT. In contrast to the MT group, only 19 subjects in the group of combined tests needed MT/surgical thoracoscopy, and injures from thoracoscopy were decreased.

## Conclusion

Overall, the clinical pathway guiding combined application of interventional pulmonology is useful for tracing the causes of undiagnosed PEs. The diagnostic yield of undiagnosed PEs increase significantly compared with that of MT alone.

## Methods

### Study population

We performed a retrospective study at a tertiary care academic medical centre. The keyword “pleural effusions” was searched in the Hospital Information System (HIS) of Dalian Municipal Central Hospital from January 1, 2012, to December 31, 2018. The eligibility criteria for PEs were as follows: (a) newly found within the duration of hospital stay; (b) accompanied main lesions within the chest; (c) met the definition of undiagnosed PEs; (d) age older than 18 years; (f) optimal diagnostic tests depending on guidance of the clinical pathway performed; (j) alternative subsequent diagnostic tests had to be completed in the same hospital stay if selected; and (h) related management followed over 6 months, including for subjects who did not undergo subsequent diagnostic tests. The exclusion criteria were as follows: (a) known PEs repeatedly treated; (b) incomplete clinical data recorded; (c) refused the clinical pathway; (d) accompanying major lesions outside the chest, with other diagnostic tests preferred; and (e) follow-up duration less than 6 months or loss to follow-up. Undiagnosed PEs was defined as causes of PEs that remained unknown after thorough examination of clinical history and chest images, PF analysis and blood tests^4^. A flow chart for enrolling clinical pathway to guide undiagnosed PEs was shown on Fig. 3. The clinical pathway guided selections of optimal and subsequent diagnostic tests by matching profitable chest lesions with respective diagnostic adaptations of IP (Fig. 1). The selective tests in clinical pathway were based on a multidisciplinary discussion and at least a radiologist, a pulmonologist, a thoracic surgeon and a clinician were included. Optimal diagnostic tests were defined as initial diagnostic tests selected depending on the guidance of the clinical pathway. Subsequent diagnostic tests were defined as further tests in subjects negative for optimal diagnostic tests within the same hospital stay. Eligible subjects were divided into a group of combined tests and a group of MT, depending on optimal diagnostic tests. Tests in the group of combined tests included common bronchoscopy (Olympus, BF-6C260, Japan), fine bronchoscopy (Olympus, BF-6C260, Japan), therapeutic bronchoscopy (Olympus, BF-1T260, Japan), EBUS-TBNA (Olympus, BF-UC260FW, Japan), guided bronchoscopy, CT-assisted TTCB and ultrasound-assisted CNB. Guided bronchoscopy included the guidance of radial endobronchial ultrasound (Olympus, UM-S20-17S, Japan), VBN (Broncus, LungPoint VBN, America) and fluoroscopy. Single MT (Olympus, LTF240, Japan) was used as the optimal diagnostic test for the MT group. As the guidance of clinical pathway, common bronchoscopy would be preferentially selected if pulmonary lesions involved or within the central bronchus, EBUS-TBNA was favoured when pulmonary lesions were adjacent to the central bronchus or with the enlarged mediastinal/hilar lymph nodes, guided bronchoscopy would be preferred if pulmonary nodules/masses were larger than 20 mm with discernible bronchus signs, CT-assisted transthoracic core biopsy was preferred if pulmonary nodules were less than 20 mm, image guided cutting needle biopsy was the recommendation if the pleural thickness was larger than 10 mm and pulmonary lesions were miliary. MT was preferred only when undiagnosed PEs was the initial symptom and pulmonary lesions were miliary or absent. Types of optimal and subsequent diagnostic tests, pathological findings, sex, age, previous clinical history, blood results, analysis of PF and clinical diagnosis were extracted and recorded for each subject.

### Diagnostic criteria

Exudative or transudative effusions were identified by Light’s criteria^6^. MPE was defined as thepresence of malignant cells in PF or newly present PEs accompanied an advanced stage of malignant tumour^10^. Tuberculous pleural effusion was diagnosed by a positive smear and/or culture of *Mycobacterium tuberculosis* in PF or granulomas, which was noted on pleural biopsy^17,33^. Non-specific pleuritis was interpreted as pleural biopsy without granulomatous inflammation or tumour cells^23,34^. Parapneumonic effusions (PPE) were exudative effusions associated with underlying pneumonia^32^. Complicated PPE indicated PPE progressing from the infective stage to the fibrinopurulent stage and requiring thoracic drainage or surgery to resolve^5,9,32^. Empyema was defined as PEs with the presence of pus or a positive culture^5^.

### Ethics approval

This retrospective study followed the ethics principles for medical research in the Declaration of Helsinki and was approved by the Dalian Municipal Central Hospital Human Ethics Committee (2020-002-01). Comprehensive informed consent was obtained from all participants or their legal guardians, and written informed consent was signed before every invasive test was performed.

The follow-up time was 3 months, and the duration was longer than 6 months for all eligible subjects. Subjects with ineffective treatment or recurrent or progressive PE during follow-up were evaluated again.

### Statistical analysis

Data were analysed by using SPSS (IBM Corporation, New York, USA). Measurement data were reported as mean values ± SD. Count data were analysed using Pearson chi-square tests. All *p* values were double-sided, and *p* < 0.05 indicated a significant difference.

## Supplementary Information

Supplementary Table 1.

Supplementary Table 2.

Supplementary Table 3.

## Data Availability

All data generated or analysed during this study are included in this published article and its supplementary information files.

## Abbreviations

CHF: Chronic heart failure
CNB: Cutting needlebiopsy
CPB: Closed pleural biopsy
EBUS-TBNA: Endobronchial ultrasound-guided transbronchial needle aspiration biopsy
ENB: Electromagnetic navigation bronchoscopey
HIS: Hospital Information System
IP: Interventional pulmonology
MPE: Malignant pleural effusion
MT: Medical thoracoscopy
NSP: Non-specific pleuritis
PF: Pleural fluid
PE: Pleural effusion
PPE: Parapneumonic effusion
TPE: Tuberculous pleural effusion
TTCB: Transthoracic core biopsy
VATS: Video-assisted thoracic surgery
VBN: Virtual bronchoscope navigation
